# Randomized SMILES strings improve the quality of molecular generative models

**DOI:** 10.1186/s13321-019-0393-0

**Published:** 2019-11-21

**Authors:** Josep Arús-Pous, Simon Viet Johansson, Oleksii Prykhodko, Esben Jannik Bjerrum, Christian Tyrchan, Jean-Louis Reymond, Hongming Chen, Ola Engkvist

**Affiliations:** 1 Hit Discovery, Discovery Sciences, R&D, AstraZeneca Gothenburg, Mölndal, Sweden; 2 Medicinal Chemistry, BioPharmaceuticals Early RIA, R&D, AstraZeneca Gothenburg, Mölndal, Sweden; 3 Department of Chemistry and Biochemistry, University of Bern, Freiestrasse 3, 3012 Bern, Switzerland

**Keywords:** Deep learning, Generative models, SMILES, Randomized SMILES, Recurrent Neural Networks, Chemical databases

## Abstract

Recurrent Neural Networks (RNNs) trained with a set of molecules represented as unique (canonical) SMILES strings, have shown the capacity to create large chemical spaces of valid and meaningful structures. Herein we perform an extensive benchmark on models trained with subsets of GDB-13 of different sizes (1 million, 10,000 and 1000), with different SMILES variants (canonical, randomized and DeepSMILES), with two different recurrent cell types (LSTM and GRU) and with different hyperparameter combinations. To guide the benchmarks new metrics were developed that define how well a model has generalized the training set. The generated chemical space is evaluated with respect to its uniformity, closedness and completeness. Results show that models that use LSTM cells trained with 1 million randomized SMILES, a non-unique molecular string representation, are able to generalize to larger chemical spaces than the other approaches and they represent more accurately the target chemical space. Specifically, a model was trained with randomized SMILES that was able to generate almost all molecules from GDB-13 with a quasi-uniform probability. Models trained with smaller samples show an even bigger improvement when trained with randomized SMILES models. Additionally, models were trained on molecules obtained from ChEMBL and illustrate again that training with randomized SMILES lead to models having a better representation of the drug-like chemical space. Namely, the model trained with randomized SMILES was able to generate at least double the amount of unique molecules with the same distribution of properties comparing to one trained with canonical SMILES.
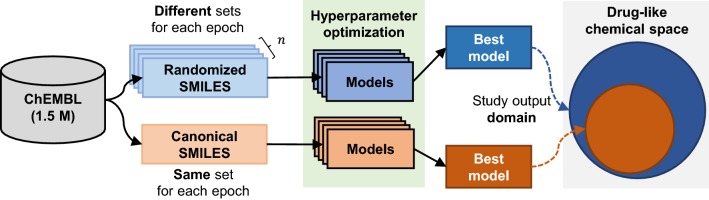

## Introduction

Exploring the unknown chemical space in a meaningful way has always been one of the major objectives in drug discovery. Given the fact that the drug-like chemical space is enormous (the lower estimation is 10^23^ molecules) [1], it cannot be easily searched. One of the most interesting attempts to understand the chemical space is the GDB project [2], which encompasses a set of databases that combinatorially enumerate large parts of the small molecule fragment-like chemical space. Currently there are databases that enumerate most fragment-like molecules with up to 13 (975 million molecules) [3] and 17 (166 billion molecules) [4] heavy atoms. Another approach, GDB4c [5], enumerates ring systems up to four rings both in 2D (circa one million ring systems) and 3D (more than 6 million structures). Although managing billion-sized databases is computationally challenging, the enumerative approach has proven useful to study the entire small drug-like molecular chemical space in an unbiased way [6].

In the last 2 years molecular deep generative models have emerged as a powerful method to generate chemical space [7] and obtain optimized compounds [8]. Given a training set with molecules (generally a database such as ChEMBL [9]), these models learn how to create molecules that are similar but not the same as those in the training set, thus spanning a bigger chemical space than that of the training data. Either after or during training, the probability of generating molecules with specific properties can be altered with techniques such as reinforcement [8] or transfer learning [7, 10]. Multiple architectures have been reported in literature: the first one is Recurrent Neural Networks (RNNs) [7], but also others such as Variational AutoEncoders (VAEs) [11], Generative Adversarial Networks (GANs) [12, 13], etc. [14]. Due to its simplicity, in most published research the format representing molecules is the canonical SMILES notation [15], a string representation unique to each molecule. Nevertheless, models that use the molecular graph directly are starting to gain interest [16, 17].

Notwithstanding the popularity of RNNs, the idiosyncrasies of the canonical SMILES syntax can lead to training biased models [18]. Specifically, models trained with a set of one million molecules from GDB-13 have a higher probability of generating molecules with fewer rings. Additionally, the canonical SMILES representation can generate substantially different strings for molecules that are very similar, thus making some of them more difficult to sample. To prove this, these models were sampled with replacement 2 billion times and at most only 68% of GDB-13 could be obtained from a theoretical maximum of 87%. This maximum would be from sampling with replacement the same number of times from a theoretical ideal model that has a uniform probability of obtaining each molecule from GDB-13, thus obtaining the least possible biased output domain.

We performed an extensive benchmark of RNN models trained with SMILES obtained from GDB-13 whilst exploring an array of architectural changes. First and foremost, models were trained with three different variants of the SMILES notation. One of them is the commonly used canonical SMILES, another one are randomized SMILES (also known as enumerated SMILES), which have been used as a data amplification technique and are shown to generate more diversity in some model architectures [19–21]. The third one is DeepSMILES [22], a recently published modification of the canonical SMILES syntax. Secondly, models were trained with decreasing training set sizes (1,000,000, 10,000 and 1000 molecules) to explore the data amplification capabilities of randomizes SMILES. Thirdly, the two most used recurrent cell architectures were compared: long short-term memory (LSTM) [23] and Gated Recurrent Unit (GRU) [24]. GRU cells are widely used as a drop-in replacement of LSTM cells with an noticeable speed improvement, but it has been shown that in some tasks they perform worse [25]. Fourthly, regularization techniques such as dropout [26] in conjunction with different batch sizes were also tested and their impact on the generated chemical space assessed. All of the benchmarks were supported by a set of metrics that evaluate the uniformity, completeness and closedness of the generated chemical space. With this approach, the generated chemical space is treated as a generalization of the training set to the entire GDB-13 and the chemical space exploration capability of the models can be assessed. Finally, to demonstrate how the same methodology can be used to train models that generate real-world drug-like compounds, models were trained with a subset of the ChEMBL [9] database.

## Methods

### Randomized SMILES strings

**Fig. 1:**
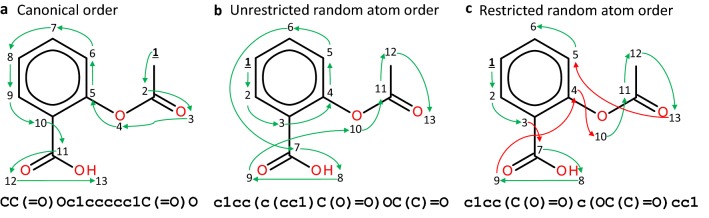
Traversal of the molecular graph of Aspirin using three methods: **a** the canonical ordering of the molecule; **b** atom order randomization without RDKit restrictions; **c** Atom order randomization with RDKit restrictions of the same atom ordering as **b**. Atom ordering is specified with a number ranking from 1 to 13 for each atom and the arrows show the molecular graph traversal process. Notice that the atom ordering is altered in **c**, prioritizing the sidechains (red arrows) when traversing a ring and preventing SMILES substrings like c1cc(c(cc1))

To obtain canonical SMILES the atoms in a given molecule have to be uniquely and consistently numbered. In the case of RDKit this is done by using a modified version of the Morgan algorithm [27, 28]. The SMILES generation algorithm is then able to traverse the molecular graph always in the same way (Fig. 1a). Some atom orderings can lead to overly complicated SMILES strings and that is why RDKit has some built-in fixes that alter atom order on-the-fly. They prevent strange combinations, such as prioritizing traversing sidechains before the ring atoms, and are by default active.

One easy way of obtaining randomized SMILES is by randomizing atom ordering. This does not alter how the algorithm traverses the graph (i.e., depth-first in the case of RDKit), but changes the starting point and in what order the branching paths are selected. With this approach, theoretically, at most $n!$ different SMILES can be generated on a molecule with $n$ heavy atoms, yet the resulting number of different combinations ends up being much lower. The two different variants of randomized SMILES used here (Fig. 1b, c) only change on the application of the RDKit fixes. This makes the unrestricted version a superset of the restricted one, which includes the SMILES that are disallowed in the regular restricted version.

### RNNs trained with SMILES

#### Pre-processing SMILES strings

SMILES strings of all variants need to be tokenized to be understood by the model. Tokenization was performed on a character basis with the exception of some specific cases. The first are the “Cl” and “Br” atoms, which are two-character tokens. Second are atoms with explicit hydrogens or charge, which are between brackets (e.g., “[nH]” or “[O-]”). Third, ring tokens can be higher than 9 in which case the SMILES syntax represents the number prepended with the “%” character (e.g., “%10”). These rules apply to all SMILES variants used in this research. Lastly, the begin token “^” was prepended and the end token “$” appended to all SMILES strings. The tokenization process was performed independently for each database and yielded vocabulary sizes of 26 in GDB-13 and 31 in ChEMBL. When training the DeepSMILES models, the official implementation [22] was used to convert the SMILES.

#### Architecture

**Fig. 2:**
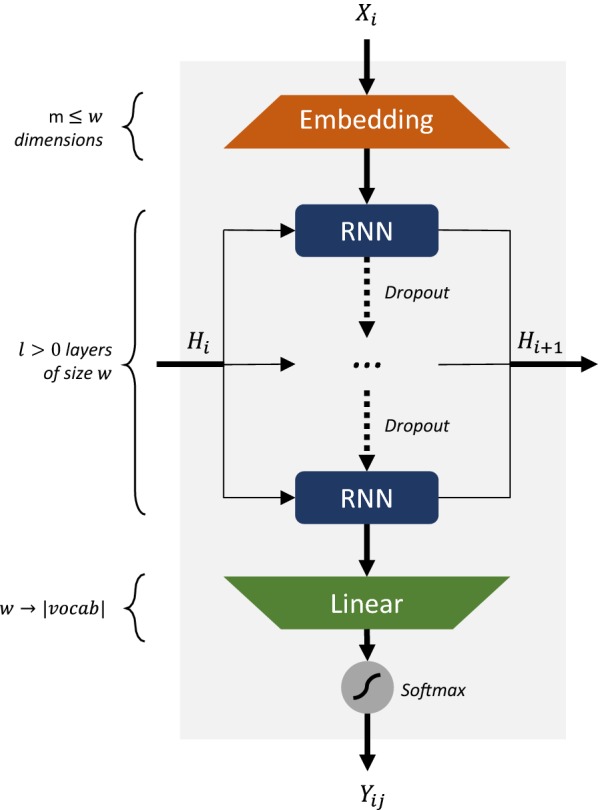
Architecture of the RNN model used in this study. For every step $i$, input one-hot encoded token $X_{i}$ goes through an embedding layer of size $m \le w$, followed by $l > 0$ GRU/LSTM layers of size $w$ with dropout in-between and then a linear layer that has dimensionality $w$ and the size of the vocabulary. Lastly a softmax is used to obtain the token probability distribution $Y_{ij}$. $H_{i}$ symbolizes the input hidden state matrix at step $i$

The model architecture used is similar to the one used in [7, 8, 18] and is illustrated in Fig. 2. The training set sequences are pre-processed, and for each training epoch the entire training set is shuffled and subdivided in $b$ batches. The encoded SMILES strings of each batch are fed token by token to an embedding layer of $m$ dimensions, followed by $l$ layers of LSTM [23] /GRU [24] cell size $w$. To prevent squeezing the encoded input, the embedding dimensions should be $m \le w$. Between the inner RNN layers there can be dropout layers [26] with a probability $d$. The output from the cells is squeezed to the vocabulary size $v$ by a linear transformation layer and a softmax is performed to obtain the probabilities of sampling each token in the next position. This is repeated for each token in the entire sequence.

#### Training a model

Following [18], all models have two sets: a training and a validation set. The validation set holds molecules that are in the target chemical space but are not used for training the model. Depending on the training set different splits can be made. In Table 1 is shown the size of the training and validation sets for each of the benchmarks (see Additional file 1: Methods S1 for more information on how the databases were filtered). In the case of models trained with randomized SMILES, a new sample of randomized SMILES of the same molecules are used for the training and validation set for each epoch. These training set files are created beforehand and the model uses a different file for each epoch. For example, a model trained with one million molecules for 300 epochs will have approximately 300 million different randomized SMILES, although the number is generally lower because some SMILES are more commonly sampled than others.

**Table 1:** Training and validation set sizes for the different benchmarks

Model	Training set size	Validation set size
GDB-13 1M	1,000,000	10,000
GDB-13 10K	10,000	1000
GDB-13 1K	1000	1000
ChEMBL	1,483,943	78,102

Notice that depending on the expected size of the target chemical space and the total amount of molecules, different ratios have been used

During each epoch the training set is shuffled and minibatches of size $b$ are created. These batches are in the form of a matrix with a row for each encoded SMILES string and appended with end tokens as padding. The “teacher’s forcing” approach is used in training, which means that the correct token is always input in the next step, regardless of the prediction from the model [29]. The loss function to minimize by the model is the average negative log-likelihood (NLL) of the entire batch of tokenized SMILES strings. Given $X_{i}$ and $x_{i}$ as the sampled and expected token at previous step $i \ge 0$ respectively and the current time step $T \ge 0$, the partial NLL of a SMILES string is computed as:

$$J\left( T \right) = NLL\left( T \right) = - \ln P\left( {X_{0} = x_{o} } \right) - \mathop \sum \limits_{t = 1}^{T} \ln P\left( {X_{t} = x_{t} |X_{t - 1} = x_{t - 1} \ldots X_{1} = x_{1} } \right)$$

To prevent instability during training, the computed gradients are updated so that the norm is $1.0$. When performing a forward-pass on a batch, the model does not apply any mask to already finished sequences. This makes the model run slightly faster because no masks are computed and, as the padding token is the end of sequence, it does not affect the quality of the training process. All weight matrices are initialized from a uniform random distribution ${\mathcal{U}}\left( { - \sqrt {1/w} ,\sqrt {1/w} } \right)$. The learning decay strategy is based on a custom metric calculated at each epoch (UC-JSD) and is discussed in section “Adaptive learning rate decay strategy” of the Additional file 1: Methods S2.

### Benchmark

The models were optimized over the hyperparameter combinations shown in Table 2. The two models with bigger training set sizes were optimized for fewer parameters, as training times were much longer. On the other hand, the two smaller models allowed for more optimizations, as each epoch took few seconds to calculate. After the first benchmark, GRU cells were dropped because of their consistently lower performance.

**Table 2:** Hyperparameter combinations used in the grid search

Model	l	w	d	b	RNN
GDB-13 1M	3	512	0, 25, 50	64, 128, 256, 512	GRU, LSTM
GDB-13 10K	2, 3, 4	256, 384, 512	0, 25, 50	8, 16, 32	LSTM
GDB-13 1K	2, 3, 4	128, 192, 256	0, 25, 50	4, 8, 16	LSTM
ChEMBL	3	512	0, 25, 50	64, 128, 256, 512	LSTM

Number of layers (*l*), dimensions of the RNN layers (*w*), dropout rate % (*d*), batch size (*b*), RNN cell type (*RNN*)

After each hyperparameter optimization, the best epoch was chosen as follows. A smoothing window function size 4 was applied to the UC-JSD calculated on each epoch, selecting the epoch with the lowest UC-JSD (see next section) as the best one.

#### UC-JSD—a metric for generative models

The metric used for the benchmark is derived from previous research [18]. There, it was hypothesized that the best models are those in which the validation, training and sampled set NLL distributions are uniform and equivalent. The Jensen–Shannon Divergence (JSD) measures the divergence between a set of probability distributions [30] and is calculated as:

1 $$JSD = H\left( {\mathop \sum \limits_{d \in D} \alpha_{i} \cdot d_{i} } \right) - \mathop \sum \limits_{d \in D} \alpha_{i} H\left( {d_{i} } \right)$$

where $H\left( d \right)$ is the Shannon entropy of a given probability distribution and $\forall d \in D; 0 < \alpha_{d} < 1$ and $\sum \alpha_{d} = 1$ are weights. The $JSD \to 0$ when $\forall d_{i} \in {\mathcal{D}};d_{i} = d_{j} ;i \ne j$, which does not explicitly consider uniformity (i.e., the distributions can be non-uniform but equal).

To solve this issue the Uniformity–Completeness JSD (UC-JSD) was designed. Instead of binning the raw distribution NLLs, each of the NLLs is used as it is. Given the three NLL vectors for the sampled, training and validation sets of the same size $NLLS = \left\{ {NLL_{validation} ,NLL_{training} ,NLL_{sampled} } \right\}$ and $\alpha_{i} = 1/3$, the values in each vector are divided by the total sum, giving a probability distribution with as many values as items in the vector. Then (Eq. 1 is used to calculate the JSD between the three distributions. Notice that, since the model is sampled randomly, the $UC_{JSD} \to 0$ either in the highly unlikely case that all the samples have molecules with the same NLL or all three distributions are uniform, and the model is complete.

#### Sampling the best epoch of a model

The main objective of sampling a model is to assess the properties of the output domain. Namely, in the case of GDB-13, the uniformity (equal probability of sampling), completeness (sampling all molecules from GDB-13) and closedness (only molecules from GDB-13 are sampled) are to be assessed. To ease the evaluation of the models, three ratios representing the three properties were defined.

Given a sample with replacement size $k$, the $valid$ (SMILES parsed correctly with repeats), $in$ (SMILES with repeats in GDB-13), $unique$ (sampled unique canonical SMILES in GDB-13) subsets are obtained. Both $ratio_{valid} = \frac{{\left| {valid} \right|}}{k}$ and $ratio_{in} = \frac{{\left| {in} \right|}}{k}$ are relative to the entire sample but $ratio_{unique} = \frac{{\left| {unique} \right|}}{{\left| {GDB13} \right|}}$ is relative to $\varphi \left( k \right)$, which represents the expected ratio of different molecules obtainable when a sample size $k$ with replacement is performed on a model that generates uniformly all molecules from and only from GDB-13 (ideal model) [18] (i.e., $\varphi \left( {2 \cdot 10^{9} } \right) = 0.8712$). This allows to define the ratios as:

$$completeness = \frac{{ratio_{unique} }}{\varphi \left( k \right)}$$

$$uniformity = \frac{{ratio_{unique} }}{{\varphi \left( {\left| {in} \right|} \right)}}$$

$$closedness = ratio_{in}$$

Also, the $UCC = completeness \cdot uniformity \cdot closedness$ was also defined as a unified score that heavily penalizes models that have low scores. See the Additional file 1: Methods S2–4 for further details on how the benchmark was performed.

### Technical notes

All the software was coded in Python 3.6.8. The models were coded using the PyTorch 1.0.1 library [31]. Unless specified, the chemistry library used throughout is RDKit 2019_03_01 [32] and for all the big data processing Spark 2.4.3 [33] was used. All plots were made with matplotlib 3.0.3 [34] and seaborn 0.9.0 [35]. The GPU hardware used to train and sample the models were Nvidia Tesla V100 (Volta) 16 GB VRAM cards using CUDA 9.1 on stable driver 390.30. The MOSES and FCD benchmarks were calculated using the code provided in (https://github.com/molecularsets/moses).

## Results

### Optimizing generative models with 1 million SMILES from GDB-13

#### Canonical vs. randomized SMILES

**Fig. 3:**
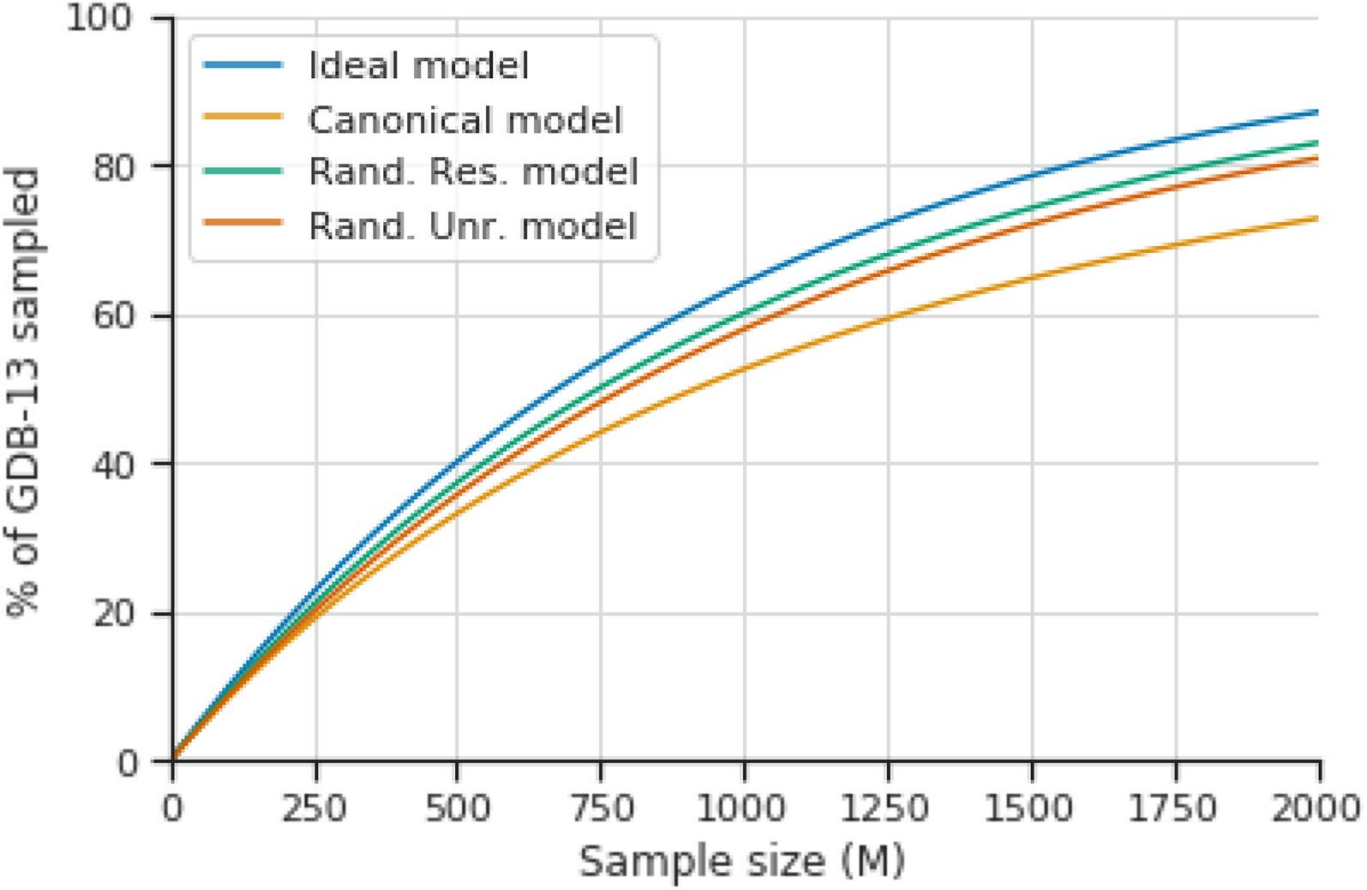
Plot illustrating the percent of GDB-13 sampled alongside the sample size of the ideal model (blue) and the best of the canonical (yellow), randomized restricted (green) and randomized unrestricted (orange) models. Notice that the ideal model is always an upper bound and eventually ($n \sim 21B$) would sample the entire GDB-13. The trained models would reach the same point much later

**Fig. 4:**
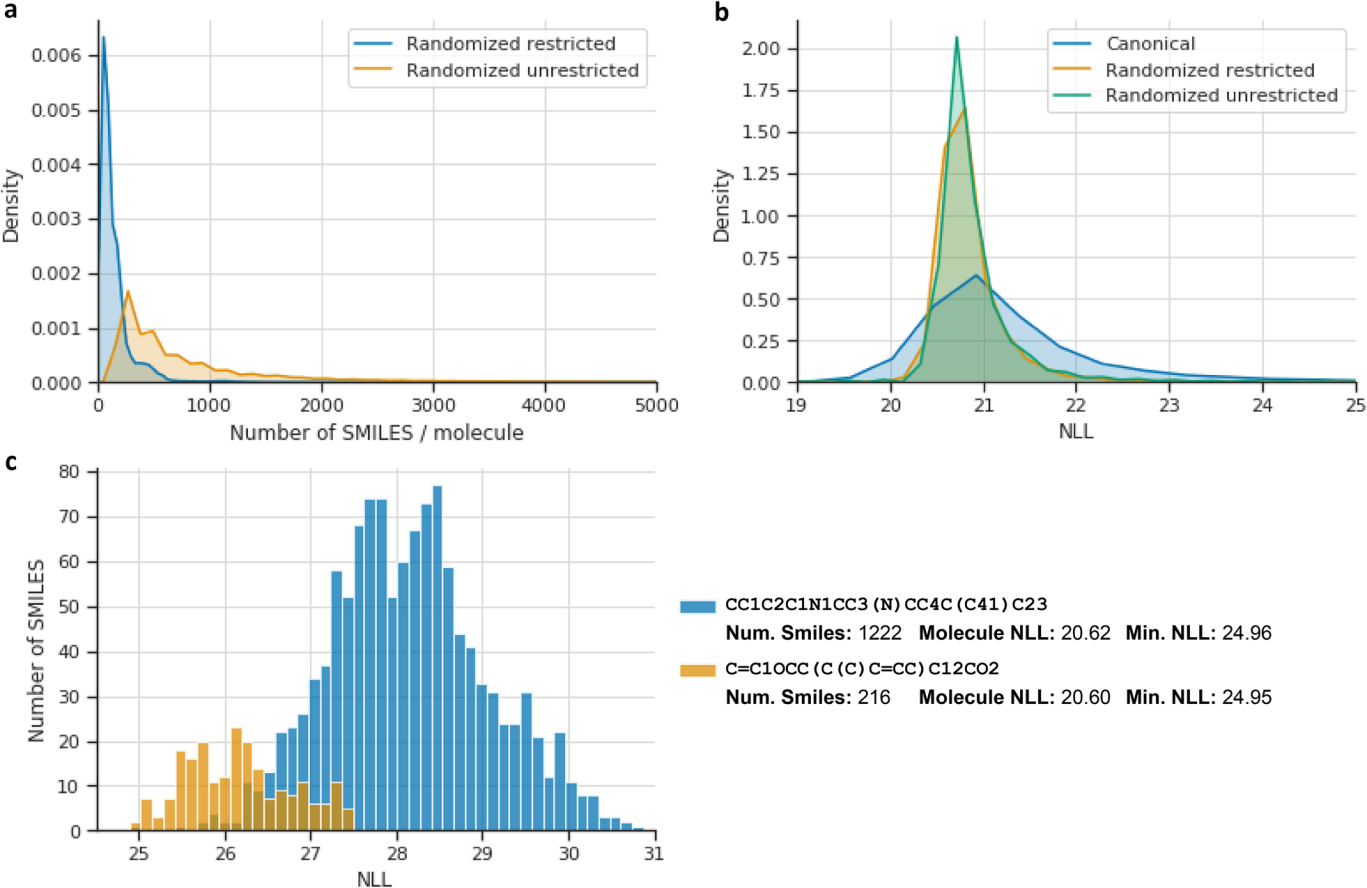
Histograms of different statistics from the randomized SMILES models. **a** Kernel Density Estimates (KDEs) of the number of randomized SMILES per molecule from a sample of 1 million molecules from GDB-13. The plot has the x axis cut at 5000, but the unrestricted randomized variant plot has outliers until 15,000. **b** KDEs of the molecule negative log-likelihood (NLL) for each molecule (summing the probabilities for each randomized SMILES) for the same sample of 1 million molecules from GDB-13. The plot is also cropped between range $\left( {19,25} \right)$. **c** Histograms between the NLL of all the restricted randomized SMILES of two molecules from GDB-13

Hyperparameter optimizations of the three main SMILES variants (canonical, randomized restricted and randomized unrestricted) were performed on models trained with 1 million molecules randomly sampled from GDB-13 (Table 2). A $k = 2 \cdot 10^{9}$ SMILES sample was performed on the best epoch for each of the models trained in the benchmark (see Additional file 1: Methods S1). Results show (Table 3, Additional file 2: Figure S4 for the best hyperparameter combinations for each SMILES type and Additional file 3: Table S1 for all results) that the randomized variants greatly outperform canonical SMILES. The best canonical SMILES model was only able to enumerate 72.8% of GDB-13 compared to the 83.0% of the restricted randomized SMILES (Fig. 3). All three metrics, uniformity, completeness and closedness are much higher and show that the restricted randomized models are theoretically able to generate most of GDB-13 with uniform probability. This can be further seen in Fig. 4b, where the NLL distribution of a sample of molecules from the GDB-13 randomized SMILES models is centered at $NLL_{GDB13} = - ln\left( {\frac{1}{{\left| {GDB13} \right|}}} \right) = 20.6$ and is much narrower than that of the canonical variant model.

**Table 3:** Best models trained on subsets of GDB-13 after the hyperparameter optimization

Set	SMILES	Time	% GDB-13	Valid	Unif	Comp	Closed	UCC
1M	Canonical	4:08	72.8	0.994	0.879	*0.836*	0.861	0.633
	Rand. unr.	31:47	80.9	0.995	0.970	0.929	0.876	0.790
	Rand. unr. no DA	1:37	77.0	0.987	0.957	0.795	0.883	0.672
	*Rand. rest.*	*7:19*	*83.0*	*0.999*	*0.977*	*0.953*	*0.925*	*0.860*
	Rand. rest. no DA	1:21	78.2	0.992	0.957	0.829	0.898	0.712
	DS branch	1:33	72.1	0.987	0.881	0.828	0.834	0.608
	DS rings	1:11	68.6	0.979	0.852	0.788	0.798	0.535
	DS both	1:05	68.4	0.979	0.851	0.785	0.796	0.532
10K	Canonical	0:04	38.8	0.905	0.666	0.445	0.426	0.126
	*Rand. rest.*	*0:36*	*62.3*	*0.974*	*0.882*	*0.715*	*0.598*	*0.377*
1K	Canonical	0:01	14.5	0.504	0.611	0.167	0.133	0.014
	*Rand. rest.*	*0:04*	*34.1*	*0.812*	*0.790*	*0.392*	*0.276*	*0.085*

See “Methods” section for a description of the ratios

Best result for each training set size are indicated in italics

*Set* Benchmark training set size, *SMILES* SMILES variant, including randomized variants with and without data augmentation (DA), *Time* training time up in hh:mm, *% GDB-13* Percent of unique molecules from GDB-13 generated in a 2 billion sample with replacement, *Valid* valid SMILES, *Unif* uniformity ratio, *Comp* completeness ratio, *Closed* closedness ratio, *UCC* UCC ratio

Comparing the two variants of randomized SMILES, models trained with both variants have a similarly uniform output domain (Fig. 4b), but models trained with restricted randomized variant have a more complete and more closed domain than those trained with the unrestricted variant. The output domain of the ideal randomized SMILES models would comprise all possible SMILES strings of any given variant possible to be generated from all molecules in GDB-13. This contrasts with the canonical model, in which the output domain is one SMILES per molecule. Each molecule has a different number of SMILES strings, depending on its topology, although only a few (generally highly cyclic or branched molecules) have numbers above 1000 (Fig. 4a). Knowing that the training objective is to obtain a uniform posterior distribution, it would be expected that molecules with more randomized SMILES should have a higher probability of being sampled than those that have fewer. However, this is never the case as models trained with randomized SMILES have a much more uniform posterior probability distribution than those trained with canonical SMILES (Fig. 4b). The model naturally learns to prioritize some SMILES in molecules with a large number of possible SMILES, and to have a more uniform distribution among all possible SMILES on molecules that have less. This can be seen in Fig. 4c, where two molecules have the same NLL, but one (blue) has six times the number of possible SMILES than the other (orange).

Models trained with randomized SMILES without data augmentation (the same SMILES strings each epoch) were also benchmarked. Results show (Table 3, Additional file 2: Figure S4 for the best hyperparameter combinations for each SMILES type and Additional file 3: Table S1 for all results) that they perform better than the models trained with canonical SMILES but worse than those with data augmentation. This indicates that not using the canonical representation constraint makes better models, but also that data augmentation has a positive impact on the training process.

DeepSMILES is a SMILES syntax variant that alters the syntax and changes how rings and branching are represented [22]. Three different forms of DeepSMILES were explored: one with the new ring syntax, another with the new branching syntax and a last one with both changes. Results show (Table 3, Additional file 3: Table S1 complete) that the performance is consistently lower than using normal canonical SMILES. The validity is generally 1–3% lower than in canonical SMILES, possibly indicating that the model has difficulties in learning the basics of the syntax.

The hyperparameter optimization also gives some hints on how dropout, batch size and cell type affect the training process, although it varies for each SMILES variant. Plots for each hyperparameter compared to the four ratios and the training time were drawn (Additional file 2: Figure S1) and show that adding dropout only makes canonical SMILES models better. The model improves its completeness, but at the expense of closedness, meaning that it generates more molecules from GDB-13 at the expense of making more mistakes. On the other hand, larger batch sizes have generally a positive impact in models of all SMILES variants and at the same time make training processes much faster. But the most interesting result is that the best models for all SMILES variants use LSTM cells. Moreover, even though the training time per epoch of the GRU cells is lower, LSTM models are able to converge in fewer epochs.

Similarity maps for the randomized SMILES were also plotted (Additional file 2: Figure S2) and confirm that models trained with randomized SMILES are able to generate mostly all molecules from GDB-13 with uniform probability. Only molecules on the left tip of the half-moon (highly cyclic) are slightly more difficult to generate, but this is because they have extremely complicated SMILES with uncommon tokens and ring closures. Additionally, maps colored by the number of SMILES per molecule were created and show that most of the molecules that have more randomized SMILES are the same as those that are difficult to sample in the canonical models.

#### UC-JSD can be used to predict the best models

**Fig. 5:**
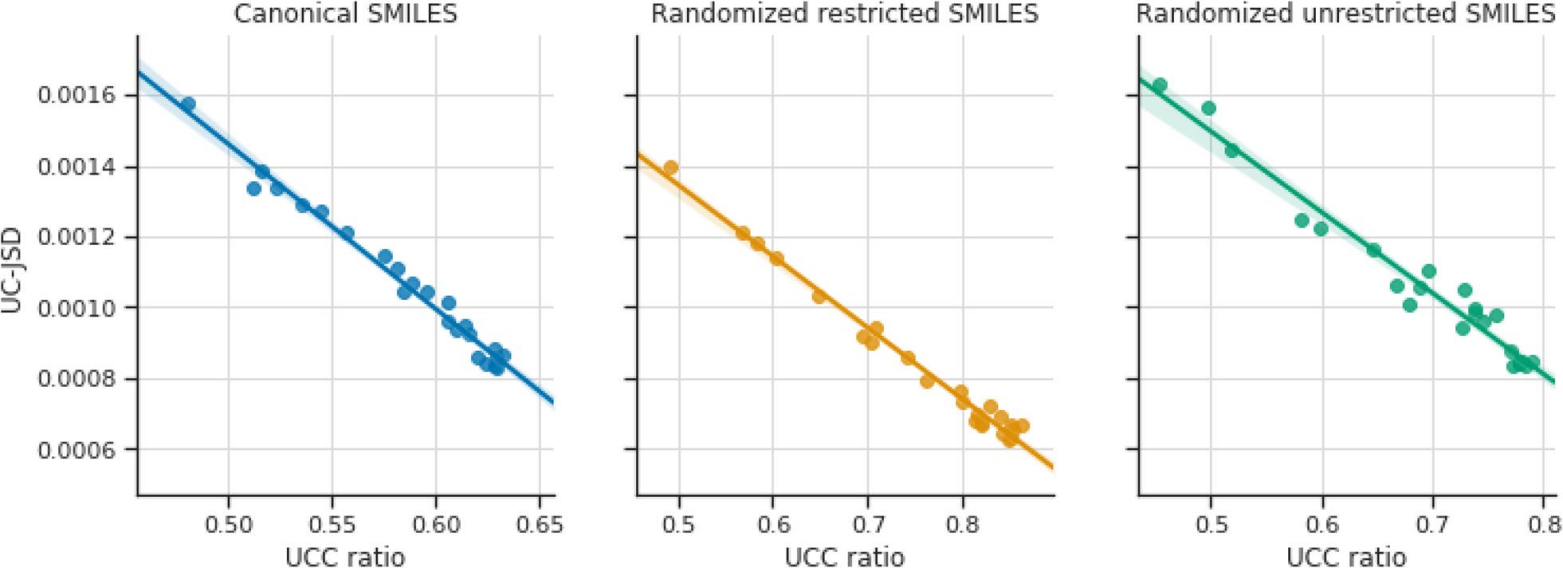
Linear regression plots between the UC-JSD and the UCC ratio. **a** Canonical SMILES $R^{2} = 0.931$. **b** Restricted randomized SMILES $R^{2} = 0.856$. **c** Unrestricted randomized SMILES $R^{2} = 0.885$

The previous benchmark employed an adaptive learning rate strategy (see Additional file 1: Methods S2) that uses the UC-JSD metric to evaluate the quality of the models and trigger a learning rate change. Moreover, the same metric was used to select the best epochs to perform a sample for each model. Plotting the UC-JSD against UCC shows a strong correlation in all three SMILES variants (Fig. 5). It is important to notice that the UC-JSD values should not be compared between models, as the output domain is different. This result shows that it is not necessary anymore to sample all models, but only the one that has the best UC-JSD. That is why for all future benchmarks only the model with the lowest UC-JSD is sampled. Moreover, the GRU cells have not shown any improvement whatsoever compared to the LSTM cells (Additional file 2: Figure S1) and the unrestricted randomized SMILES variant performs worse than the restricted variant. Henceforth, only the restricted variant of randomized SMILES and LSTM cells will be used for the next benchmarks.

### Training generative models with smaller training sets

To further show the data augmentation capabilities of randomized SMILES, two models were trained with 1000 and 10,000 molecules respectively, randomly obtained from GDB-13. Hyperparameter optimization was modified to accommodate smaller training sets and, as models were faster to train, different network topologies were tested (Table 2). When the training sets are so small, models are often unable to learn the syntax properly and thus generate more invalid structures. The model using 1000 molecules was the most affected by this problem, with some models not even reaching 50% validity. This impacts the accuracy of the UC-JSD, because all molecules tend to have a sampling probability $p \to 0$. This makes the UC-JSD have low values because all molecules have very similar probability. For this reason, only models that had more than 50% valid SMILES were considered.

Results show (Table 3, Additional file 3: Table S1 complete) that models trained with randomized SMILES have better performance than those trained with canonical SMILES. In the models trained with 1000 molecules, those with canonical SMILES are at most able to generate up to 70% valid SMILES, although the best model was only able to generate 50% valid SMILES. Moreover, the completeness ratio of the best model is only 0.1325, meaning that most of the SMILES generated are not part of GDB-13: they correspond to molecules containing features excluded from GDB-13 (e.g. strained rings, unstable functional groups, wrong tautomer). Alternatively, the models trained with randomized SMILES show a much better behavior. Most models learn how to generate SMILES strings correctly (validity over 80%), completeness is much higher (0.2757) and their posterior distribution is more uniform. This is further illustrated with the fact that randomized SMILES models generate up to 34.11% of unique GDB-13 molecules and canonical models only 14.54%.

Models trained with a bigger sample of 10,000 molecules show similar trends but have much better performance in both cases. In this case, a model trained with randomized SMILES is able to uniquely generate 62.29% of GDB-13 while only training with less than 0.001% of the database, whereas a canonical SMILES model is only able to generate 38.77%. Closedness is much better in both models: canonical SMILES models have at most 0.4262, whereas randomized SMILES models up to 0.5978. Lastly, a large number of SMILES generated are not included in GDB-13, meaning that the model, even though generating valid molecules, does not fully learn the specific idiosyncrasies of GDB-13 molecules and generates valid molecules that break some condition.

### Improving the existing ChEMBL priors with randomized SMILES

The same benchmark study was also performed on models with a drug-like training set from ChEMBL (see Additional file 1: Methods S1 for more information on how the training set was obtained). A different and reduced set of hyperparameter values were used due to long training times (Table 2). The best models for both the canonical and restricted randomized SMILES benchmarks were obtained using the same procedure as before and a 2 billion sample was performed. Results show (Table 4, extended results Additional file 3: Table S2) that the output domain of the canonical model is much smaller than that of the randomized SMILES model. Specifically, the randomized SMILES model can generate at least twice the number of different molecules than the canonical. Nevertheless, the Fréchet ChemNet Distance (FCD) [36] between the validation set and a sampled set of 75,000 SMILES is lower on the canonical SMILES model. This could mean that the molecules generated by the canonical model have more similar properties than ChEMBL molecules, but it could also mean that the canonical model overfits and generates molecules that are similar to the training set given that the validation set and the training set are biased the same way (i.e., they are both obtained from a biased sample of the entire drug-like chemical space).

**Table 4:** Best models from the ChEMBL benchmark for both SMILES variants

SMILES	Time	% Valid	% Unique	FCD
Canonical	131:32	98.26	34.67	0.0712
Rest. Random.	84:22	98.33	64.09	0.1265

*SMILES* SMILES variant, *Time* time used to train the model hhh:mm, *% Valid* Percent of valid molecules, *% Unique* Percent of unique molecules in a 2 billion SMILES sample, *Fréchet* ChemNet Distance (FCD) between the validation and a sample of 75,000 molecules (FCD)

**Fig. 6:**
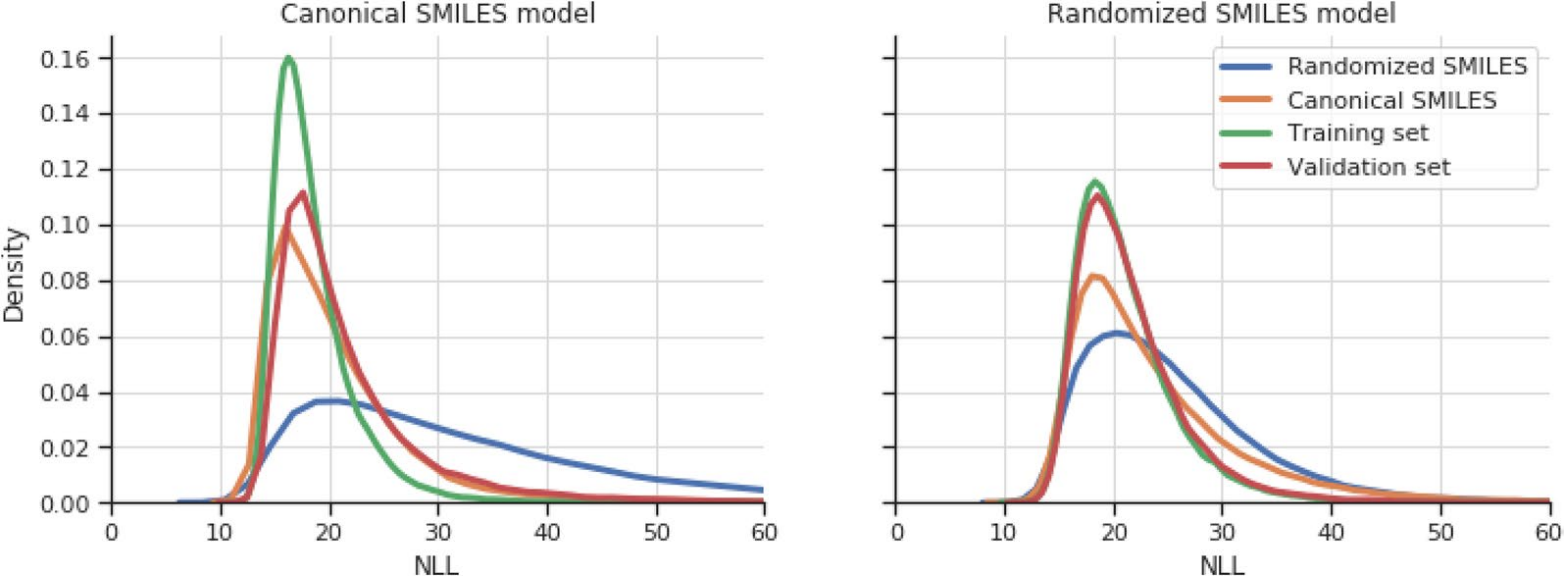
Kernel Density Estimates (KDEs) of the Molecule negative log-likelihoods (NLLs) of the ChEMBL models for the canonical SMILES variant (left) and the randomized SMILES variant (right). Each line symbolizes a different subset of 50,000 molecules from: Training set (green), validation set (orange), randomized SMILES model (blue) and canonical SMILES model (yellow). Notice that the Molecule NLLs for the randomized SMILES model (right) are obtained from the sum of all the probabilities of the randomized SMILES for each of the 50,000 molecules (adding up to 320 million randomized SMILES), whereas those from the canonical model are the canonical SMILES of the 50,000 molecules

To prove that the molecules sampled from the randomized SMILES model are at least as diverse as those in the canonical, several physicochemical properties and metrics (as used in the MOSES benchmark [37]), such as molecular weight, *logP*, Synthetic Accessibility Score (SA) [38], Quantitative Estimate of Drug-likeness Score (QED) [39], Natural-Product likeness score (NP) [40] and Internal Diversity (cross-molecule Tanimoto similarity on ECFP4) were calculated for a sample of the training, validation, randomized SMILES model and canonical SMILES model (Additional file 2: Figure S3). All of the plots are nearly identical, showing that there is no clear difference between molecules in any of the four sets. Additionally, molecule NLL plots for the same four samples were calculated for both models (Fig. 6) and show that the canonical model greatly overfits the training and validation sets compared to the randomized SMILES model, which has mostly the same distribution for both sets. When comparing the two samples, the canonical model has much lower probabilities of generating most of the molecules generated by the randomized SMILES model, but not the opposite. The randomized SMILES model is able to generate the canonical SMILES model molecules with higher likelihood than average, implying that the output domain of the canonical SMILES model is a subset of the randomized SMILES model output domain.

## Discussion

### Why are randomized SMILES better?

A SMILES molecular generative model learns by finding patterns in the SMILES strings from the training set with the goal of generalizing a model that is able to obtain all the SMILES in the training set with the highest possible probability. The procedure is exactly the same with any SMILES variant, the only thing that changes is the string representation of each molecule and, in the case of randomized SMILES, the number of different representations each molecule has. When the canonical representation is used, the model learns to generate one linear representation of each molecule obtained through a canonicalization algorithm. This means that the model must learn not only to generate valid SMILES strings, but also to generate those in the canonical form. As shown in “Methods” section (Fig. 1), the canonicalization algorithm in RDKit does not only traverse the molecule using a fixed ordering, but also adds some restrictions on how to traverse rings. Moreover, models tend to see the same patterns repeatedly, leading to premature overfitting (Fig. 6). Alternatively, randomized SMILES models do not have the canonical form limitation and can learn the SMILES syntax without restriction. When no data augmentation is used, randomized SMILES still perform substantially better than canonical SMILES. Additionally, heavy regularization with dropout in canonical models gave a better overall performance, but opposite results were obtained with randomized SMILES, showing that using different randomized SMILES on each epoch also serves as a regularization technique.

Another way of understanding why randomized variants are better is to draw a parallel with image classification models. For example, when an image classification model is trained to predict whether an image depicts a cat, the model performance can be improved with a training set that has examples of cats from all the possible angles and not always a front picture. This is not always easy to obtain in image predictive models, but in the case of molecular generative models it is extremely easy to generate *snapshots* of the same molecule from different angles (i.e., different ways of writing the SMILES string). This allows models to better learn the constraints of the training set chemical space (i.e., in the case of GDB-13: heteroatom ratios, allowed functional groups, etc.). Nevertheless, for each molecule there is a different number of randomized SMILES (Fig. 4), thus possibly generating a bias towards the molecules that have more representations. None was detected in this study possibly because larger and highly branched molecules, which tend to have more combinations, are also generally more difficult to sample and can, in effect, counteract the bias (Fig. 4c). Lastly, the restricted variant of randomized SMILES performed best, indicating that restricting the randomized SMILES algorithm makes the model generalize better. For example, the unrestricted randomized SMILES can represent the phenyl ring of aspirin (Fig. 1) in a much more convoluted way “c1cc(c(cc1)”, something that would be impossible in the restricted variant. Finding variants that perform even better should be a future research goal in this field.

### Understanding diversity in molecular generative models

A challenge in Computer-Assisted Drug Design (CADD) is to computationally generate or evaluate molecules that fit a given set of constraints. This process is not devoid of error: for instance, an inactive molecule can be predicted as active (false positive) or an active one can be predicted as inactive (false negative). From a drug design perspective, false positives are more damaging due to the economic impact a wrong prediction can have. False negatives do not impact as directly but are important nonetheless: the next *blockbuster* could be any molecule wrongly skipped by computational solutions.

Analogously, the same problem can be brought to generative models. A model can generate molecules that are outside of the target chemical space (false positives) or the output domain can collapse [41] not being able to generate a chunk of the expected chemical space (false negatives). This is very easy to assess when training models that generate the GDB-13 chemical space. First, any molecule sampled not included in GDB-13 is a false positive (closedness). It was previously shown [18] that the vast majority of these clearly do not comply to one or more conditions of GDB-13, such as having invalid functional groups, molecular graph or not being the most stable tautomer. Alternatively, any molecule comprised in GDB-13 not possible to being sampled (i.e. very high NLL) becomes a false negative (completeness). In both cases this means that the model is not able to learn correctly the rules used in the enumeration process. When canonical and randomized SMILES models are compared, the results show that randomized SMILES models perform substantially better in both properties (Table 3). They are able to learn better the filters used in enumerating GDB-13 and thus prevent the generation of incorrect molecules and at the same time generate more difficult outliers that comply with GDB-13 (Additional file 2: Figure S1, left tip of the NLL similarity maps).

Training molecules on unknown target chemical spaces is a much more difficult task. Compared to GDB-13, where the generated molecules can be checked whether or not they form part of it, there is no way of bounding the limits (if there are any) of a drug-like space. This makes benchmarking models much more complex. For instance, a model could generate an extremely diverse set of molecules, most of which are completely unrelated to the training set chemical space, compared to a model that generates less diverse and fewer molecules that are more akin to the training set chemical space. As it is unknown which is the target chemical space, assessing which is the best model is impossible. For this reason, some methods were published [37, 42] that aggregate a set of metrics to obtain a better overview of the output domain of the model. Unfortunately, they compare the models with a test set split from the training set and this tends to benefit models that overfit. Additionally, they are not able to measure mode collapse the same way as with the GDB-13 benchmark, as can be seen in [43]. This means that models may seem extremely diverse when being sampled a few thousand times, but when being sampled more times the same molecules start appearing repeatedly. This is the case with the ChEMBL models trained here. We know that the drug-like chemical space is huge [44], so we would not expect the model to collapse early. Results show that those trained with randomized SMILES have a much larger output domain (at least double) than those trained with canonical SMILES. Moreover, sets of molecules generated are physicochemically almost indistinguishable (Additional file 2: Figure S3) from sets generated from the canonical SMILES model, meaning that they are from the same chemical space. This showcases how models trained with randomized SMILES are able to represent chemical spaces that are more complete and at least as closed as those generated by models using canonical SMILES.

### SMILES generative models as action-based generative models

The most common way of understanding SMILES generative models is as grammar-based models that generate SMILES strings that are similar to the training set [7, 8], akin to language generative models [45]. Alternatively, SMILES generative models can be also understood as action (or policy)-based graph generative models [16, 46] in which a molecular graph is built stepwise. In these models, each step an action is chosen (“*add atom*”, “*add bond*”, etc.) and is sampled from a fixed or varying size action space (or policy) that has all possible actions (even invalid ones) alongside the probability of each happening. A parallelism can be partially drawn for SMILES generative models: the vocabulary is the action space in which atom tokens (“C”, “N”, “[O-]”, etc.) are “*add atom”* actions, the bond tokens (“=”, “#”, etc.) are “*add bond”* actions as are also the ring and branching tokens. The main difference is that “*add atom”* actions are always adding the new atom to the last atom added, the bond tokens add a bond to an unknown atom, which is specified just after, and the ring and branching tokens add also bonds and enable the model to jump from one place to another. Moreover, a single bond is by default added if no bond is specified between atoms when at least one is aliphatic, and an aromatic bond is added otherwise.

One of the main issues with graph generative models is that the action space can grow dangerously large, making it very challenging to train models that generate big molecules [46]. This is not the case of SMILES generative models, as they only have to choose every epoch among a limited number of options (i.e., the vocabulary). On the other hand, SMILES models traverse the graph in a very specific way, they do not allow as many options as graph models. This is specially the case with canonical SMILES: Morgan numbering greatly reduces the possible paths, as it tends to prioritize starting in sidechains rather than in the rings of the molecule [28]. This makes sense when grammatically simpler SMILES strings are desired. We think that when using randomized SMILES, models become more action-based rather than grammar-based. Additionally, this may also indicate why the syntax changes added in DeepSMILES have a detrimental effect on the learning capability of SMILES generative models, as they give the model a more complex action space. For instance, the ring token altered behavior makes the ring closures extremely grammar sensitive and the new branching token behavior makes the SMILES strings unnecessarily longer without any appreciable improvement. We think that the SMILES syntax is, with all its peculiarities, an excellent hybrid between action-based and grammar-based generative models and is, to our knowledge, the most successful molecular descriptor for deep learning based molecular generation available so far.

## Conclusions

In this research we have performed an extensive benchmark of SMILES-based generative models with a wide range of hyperparameters and with different variants of the SMILES syntax. To guide the benchmark a new metric, the UC-JSD, based on the NLL of the training, validation and sampled sets was designed. Our study shows that training LSTM cell-based RNN models using randomized SMILES substantially improves the quality of the generated chemical space without having to change anything in the generative model architecture. In the case of models trained with a sample of 1 million GDB-13 molecules, the best models are able to generate almost all molecules from the database with uniform probability and generating very few molecules outside of it. Using smaller training set sizes (10,000 and 1000) further highlights the data augmentation effect of randomized SMILES and enables training models that are able to generate 62% of GDB-13 with only a sample comprising 0.001% of the database. When training models on a ChEMBL training set, randomized SMILES models have a much bigger output domain of molecules in the same range of physicochemical properties as the canonical SMILES models. Moreover, randomized SMILES models can easily generate all molecules of the canonical SMILES output domain. The randomized SMILES variant that gave the best results is the one that has restrictions, compared to the one that is able to generate all possible randomized SMILES for each molecule. Regarding different RNN hyperparameters and architectures, we wholeheartedly recommend using LSTM cells instead of GRU, due to their improved learning capability. Nevertheless, dropout and batch size have varying behavior on each training set, thus we would recommend performing a hyperparameter optimization to obtain the best values. We envision that randomized SMILES will play a significant role in generative models in the future and we encourage researchers to use them in different model architectures and problems, such as classification and prediction models.

## Supplementary information

**Additional file 1.** Supplementary methods.

**Additional file 2.** Supplementary figures.

**Additional file 3.** Supplementary tables.

## Data Availability

The code used to train and benchmark all SMILES generative models is available in the (https://github.com/undeadpixel/reinvent-randomized) repository. The GDB-13 database is available through the Reymond group website (http://gdb.unibe.ch/downloads).

## Abbreviations

ADAM: Adaptive Moment Estimation
CADD: Computer-Assisted Drug Design
FCD: Fréchet ChemNet Distance
GAN: Generative Adversarial Network
GDB: Generated Database
GRU: Gated Recurrent Unit
HSV: Hue–Saturation–Value
JSD: Jensen–Shannon Divergence
LSTM: long short-term memory
NLL: negative log-likelihood
PCA: principal component analysis
RNN: Recurrent Neural Network
SMILES: Simple Molecular Input Line Entry System
UCC: Uniformity–Completeness–Closedness Ratio
UC-JSD: Uniformity–Completeness JSD
VAE: Variational Autoencoder
