# Supplementary material for: Randomized SMILES strings improve the quality of molecular generative models
Source: J Cheminform. 2019 Nov 21;11:71. doi: 10.1186/s13321-019-0393-0 (PMC6873550; doi:10.1186/s13321-019-0393-0)
Supplement: Supplementary file 1 — Additional file 1. Supplementary methods. [file 13321_2019_393_MOESM1_ESM.pdf]

Additional methods

Randomized SMILES strings improve the quality of molecular generative models

Josep Arús-Pous§⊥*, Simon Johansson§, Oleksii Prykhodko§, Esben Jannik Bjerrum§, Christian Tyrchan‖, Jean-Louis Reymond⊥, Hongming Chen§, Ola Engkvist§

§ Hit Discovery, Discovery Sciences, R&D, AstraZeneca Gothenburg, Sweden

‖ Medicinal Chemistry, BioPharmaceuticals Early RIA, R&D, AstraZeneca, Gothenburg, Sweden

⊥ Department of Chemistry and Biochemistry, University of Bern, Freiestrasse 3, 3012 Bern, Switzerland.

* Corresponding author: *josep.arus@dcb.unibe.ch*

S1. Databases

The GDB-13 database [1] was obtained from the official website.[1] The SMILES of the database were sanitized and canonicalized using RDKit [2] and those that gave errors during the process were discarded. This yielded a total of 975,820,187 canonical SMILES strings. All the training and validation sets were uniformly randomly sampled.

The ChEMBL 25 database [3] was also used to train models. It was obtained from the official website[2] and further cleaning was applied as follows: First, using the MolVS 0.1.1 library [4], all the molecules were sanitized, duplicates were removed, stereochemistry was removed, salts and all fragments except for the biggest one were removed and the canonical tautomer was obtained (see MolVS documentation[3]). Then, all molecules containing heavy atom types other than (C, N, O, S, Cl, Br, F) were removed. After, a series of filters were applied sequentially to remove outliers (See Table 1). To filter outliers, a histogram was plotted for each of the descriptors used and, in the case of discrete descriptors, only values with more

[1] http://gdb.unibe.ch/downloads/
[2] https://www.ebi.ac.uk/chembl/
[3] https://molvs.readthedocs.io/en/latest/

than 0.05% of the total molecules in the set were kept. In continuous descriptors an arbitrary cut-off was set. Lastly, canonical SMILES were tokenized (see next section) and some string-based filters were applied. First, SMILES with too many tokens were removed. Then, SMILES with a ratio of non-atom tokens higher than 2 were also removed. This filtered out molecules with too much branching. Finally, any non-ring token that appeared in less than 0.05% of the molecules was removed. This accounted for 26 tokens that appear seldom. The final database size was 1,562,045 compounds.

Filter	*Range allowed*	*Size after*
Full ChEMBL 25	-	1,870,461
Standardization	MolVS complete sanitization and filtered molecules with heavy atoms other than C, N, O S, Cl, Br and F.	1,647,440
Heavy atom count	$8 \leq \mathrm{HAC} \leq 70$	1,619,246
Ring count (SSSR)	$\mathrm{RC} \leq 10$	1,618,427
Size of largest ring	$\mathrm{SLR} \leq 8$	1,597,267
# C atoms / HAC	$\mathrm{ratio} \geq 0.5$	1,590,609
# tokens	$\mathrm{NT} \leq 91$ (used 0.1% cut-off)	1,568,307
# tokens / HAC	$\mathrm{ratio} \leq 2.0$	1,564,030
Token filter	Removed all non-ring tokens with less than 0.05% canonical SMILES strings.	1,562,045

Table 1: *Filters applied to ChEMBL 25 database in order (from top to bottom). The first entry is not a filter but represents ChEMBL 25 initial state. A cut-off of 0.05% was used in all the histograms (unless specified) to obtain the ranges for discrete filters. In the case of ratios, an arbitrary cut-off was set.*

S2. Benchmark

Adaptive learning rate decay strategy

The adaptive strategy used in all trained models is based on exponential learning rate decay. A parameter $0 < \gamma < 1$ is multiplied after each epoch by the previous learning rate, thus reducing it. In this approach, γ is multiplied by the previous learning rate if the average UC-JSD from the last a epochs is not lower than the current one. Additionally, the learning rate is not reduced in the first p epochs after a change (i.e., patience). This allows models to continue

to train with the same learning rate given that the model is still improving while still being resilient to training instability.

The following parameters were used in all models: $\gamma = 0.8$, $a = 4$ and $p = 8$. The ADAM optimizer [5] was used throughout with default parameters $\beta_1 = 0.9$, $\beta_2 = 0.999, \epsilon = 10^{-8}$, the starting learning rate was established at $lr_{start} = 5 \cdot 10^{-4}$ and the end learning rate was set to $lr_{end} = 10^{-5}$. These values were obtained experimentally.

Obtaining statistics for each model

Following [6], the GDB-13 models were sampled 2 billion times at the best epoch. Due to the computational costs of performing the sampling with several models and calculating the statistics, only one sample for each model was performed. Confidence intervals were obtained by calculating the expected variance when sampling the ideal model (see section "The statistical properties of the uniform model" in the Suppl. Methods).

Molecule NLL of randomized SMILES models

To be able to compare the canonical and randomized SMILES models, the NLLs of the randomized SMILES have to be normalized. To achieve that, r different randomized SMILES are calculated on each molecule and then repeats are removed. The NLL is calculated in the randomized SMILES model for each of the randomized SMILES of the molecule. Then, for each molecule, all randomized SMILES are grouped, converted back to probabilities and summed. The NLL is calculated back from the cumulative probability of each molecule, which is a lower bound approximation of the real probability of sampling a given molecule whose error depends on r.

S3. Similarity maps

Similarity maps were based on previous literature [7]. Molecular Quantum Number (MQN) [8] fingerprints were calculated using the JChem Library 18.22.0 from ChemAxon[4] for all molecules in two sets randomly sampled from GDB-13, one with 25 million molecules and

[4] http://www.chemaxon.com/

another with 1,000 molecules, also called probes. Next, the Manhattan distance (i.e., city block distance) was calculated between all molecules from the first set and the probes yielding a matrix with 25 million rows and 1,000 columns. Then, the means μ_j and standard deviations σ_j were calculated among all columns in the matrix and the normal distribution survival function $d'_{ij} = sf(d_{ij}, \mu_j, \sigma_j)$ to each distance in the matrix, thus normalizing them to the interval $[0,1]$. Then the procedure was the same as with the maps described in previous literature [6, 9]. A Principal Component Analysis (PCA) was performed to the unnormalized and unstandardized data, the first two dimensions were selected, and the 25 million molecules were binned given a rectangular viewport (w, h). Each bin represents a pixel in the plot. A descriptor was calculated for the values in each bin and coloured using Hue-Saturation-Value (HSV). The saturation was fixed at 1.0, the hue, which depends on the range of the descriptor, ranges from blue to magenta, and $value = \min(0.25, \log_{10}(\,count_{norm}))$, where $count_{norm}$ is the number of molecules in a bin normalized to $[0, 1]$.

S4. The statistical properties of the uniform model

Deriving an expression for the variance of the coverage

By treating the space of GDB-13 as the only valid SMILES, the task of regenerating the database can be simplified to a problem of sampling with replacement. Let $n \in \mathbb{Z}^+$ be the size of the total possible sample space and $k \in \mathbb{Z}^+$ be the sample size. We now enumerate the sample space and define the random variable $X \coloneqq \{X_1, \dots, X_n\}$, $X_i = 1$ if the observed sample contains compound i and 0 otherwise. Let the probability p_i denote the probability of sampling the respective compound i. Then for a sample of size k, the probability that none of the sampled compounds is compound i is $(1 - p_i)^k$, yielding the reciprocal probability, which represent the probability of sampling i to be $1 - (1 - p_i)^k$. Using the linearity property of expectation, the expected number of unique compounds sampled in k samples becomes

$$E[X] = E\left[\sum_{i=1}^{n} X_i\right] = \sum_{i=1}^{n} E[X_i] = \sum_{i=1}^{n} (1 - (1 - p_i)^k)$$

(Equation 1)

It has been proven by [6] that the expected value is maximal when the distribution is uniform, yielding the following expectation for the number of unique compounds sampled in the optimal model:

$$E[X] = n\left(1 - \left(1 - \frac{1}{n}\right)^k\right) = n\left(1 - \left(\frac{n-1}{n}\right)^k\right)$$

(Equation 2)

By the definition of variance, $Var(X) = E[X^2] - E[X]^2$, we observe that the first term $E[X^2]$ can be obtained by:

$$E[X^2] = E\left[\left(\sum_{i=1}^{n} X_i\right)^2\right] = E[nX_1^2 + n(n-1)X_1X_2] = nE[X_1] + n(n-1)E[X_1X_2]$$

(Equation 3)

where $E[X_1X_2]$ is the probability that any arbitrarily chosen pair of compounds have both members present in the sample. Note that $E[nX_1^2] = nE[X_1]$, given that both $X_1^2$and X_1 can only take the values $\{0, 1\}$, and this always happens at the same time. Using the inclusion-exclusion principle, we can express $E[X_1X_2]$ by:

$$E[X_1X_2] = 1 - 2\left(\frac{n-1}{n}\right)^k + \left(\frac{n-2}{n}\right)^k$$

(Equation 4)

Inserting, we get the expression for the variance of total number of compounds sampled.

$$Var(X) = n\left(1 - \left(1 - \frac{1}{n}\right)^k\right) + n(n-1)\left(1 - 2\left(\frac{n-1}{n}\right)^k + \left(\frac{n-2}{n}\right)^k\right) - n^2\left(1 - \left(1 - \frac{1}{n}\right)^k\right)^2$$

(Equation 5)

Since the coverage as a fraction of the total number is the desired metric, we divide $E[X]$ and $Var(X)$ by n and n^2, respectively.

Proof that the variance is maximal when the model is uniform

Since $|X| \geq 1$ (at least one unique compound must be generated for any sample of size $k > 1$), we note that both $E[X^2], E[X]^2$ are monotonically increasing functions w.r.t $E[X]$ on this domain, which for $E[X]^2$ holds by the definition of a quadratic function and, since $X_i \in \{0,1\}$, $E[X^2] = nE[X] + \sum_{i \neq j} E[X_i X_j]$, where the first term is equal to $E[X]$ and the second term is at least non-negative. Furthermore, observe that by the definition of $E[X^2]$, $E[X^2] > E[X]^2$, as $n > E[X]$ for any finite sample size k. In fact, it can be shown that $E[X^2]$ always grows at least as fast as $E[X]^2$, by differentiating with respect to $E[X]$:

$$\frac{dE[X]^2}{dE[X]} = 2E[X]$$

$$E[X^2] = n\left(1 - \left(1 - \frac{1}{n}\right)^k\right) + n(n-1)\left(1 - 2\left(\frac{n-1}{n}\right)^k + \left(\frac{n-2}{n}\right)^k\right)$$

$$= nE[X] + n(n-1)\left(\left(1 - \left(\frac{n-1}{n}\right)^k\right) + \left(1 - \left(\frac{n-1}{n}\right)^k\right) + \left(\frac{n-2}{n}\right)^k - 1\right)$$

$$= nE[X] + 2n(n-1)E[X] + n(n-1)\left(\left(\frac{n-2}{n}\right)^k - 1\right)$$

$$\frac{dE[X^2]}{dE[X]} = n + 2n(n-1) = 2n^2 - n$$

(Equation 6)

Note that, as $k \to \infty, E[X] \to n$, thus the inequality $\frac{dE[X^2]}{dE[X]} > \frac{dE[X]^2}{dE[X]}$ holds for any sample size k when $2n^2 - n > 2n$, which is true $\forall n > \frac{3}{2}$ (also for negative n, but since our choices for the size of sample space are strictly positive integers, we ignore this solution).

A corollary to this result is that the variance grows monotonically w.r.t $E[X]$ for any practical choice of n. Using the fact that $E[X]$ is maximal for the uniform model, also implies that the variance is maximal, and we get the desired result. ■

Example of using the variance of the uniform model to estimate variance of model coverage for GDB-13

Using the derived expression for the variance for a uniform model, we can use it as an upper bound for the variance of the real models. In particular, we can use this to construct confidence intervals for our sampling results. Let C denote the coverage of a model with the confidence interval computed by

$$P\left(\mu_C - Z_\alpha \cdot \frac{\sigma_C}{\sqrt{\nu_s}} \leq C \leq \mu_C + Z_\alpha \cdot \frac{\sigma_C}{\sqrt{\nu_s}}\right) = 1 - \alpha$$

(Equation 7)

where $1 - \alpha$ is a desired confidence level and ν_s the degrees of freedom. Here, Z_α is computed using Student's t-distribution with one degree of freedom. For two different models with corresponding sampled model coverages $C_1 > C_2$, let us consider a conservative estimate. Assume that we have performed just two samples for each model, $\nu_{s_1} = \nu_{s_2} = 1$. Since the true mean model coverages are unknown, we look at the worst edge case scenario, where C_1 would have been sampled from the upper bound of some confidence interval, and C_2 from the lower bound of another interval. To ensure that, even in this worst case, the confidence intervals will not overlap the difference between the sampled coverage is expressed by

$$C_1 - C_2 \geq 2Z_\alpha\sigma_1 + 2Z_\alpha\sigma_2$$

(Equation 8)

However, the true values of σ_1, σ_2 are unknown, and thus we use the upper estimation σ^* from the uniform model. Thus, if the difference in coverage between the models fulfill

$$C_1 - C_2 \geq 4Z_\alpha\sigma^*$$

(Equation 9)

we know that there is a statistical significance between the models at confidence level $1 - \alpha$. Using this threshold, given $n = 975{,}820{,}187$, $k = 2 \cdot 10^9$ and $\alpha = 0.001$, the values $\sigma^* = 8.952 \cdot 10^{-6}$ and $Z_\alpha = 3.183 \cdot 10^2$, resulting in an upper estimate of significant difference in model coverage of 0.28 %.

References

1. Blum LC, Reymond JL (2009) 970 Million druglike small molecules for virtual screening in the chemical universe database GDB-13. J Am Chem Soc 131:8732–8733. https://doi.org/10.1021/ja902302h

2. Landrum G (2014) RDKit: Open-source cheminformatics. http://www.rdkit.org/

3. Gaulton A, Hersey A, Nowotka ML, et al (2017) The ChEMBL database in 2017. Nucleic Acids Res 45:D945–D954. https://doi.org/10.1093/nar/gkw1074

4. Swain M, JoshuaMeyers (2018) mcs07/MolVS: MolVS v0.1.1. https://doi.org/10.5281/ZENODO.1217118

5. Kingma DP, Ba J (2014) Adam: A Method for Stochastic Optimization. In: Proc. ICLR

6. Arús-Pous J, Blaschke T, Ulander S, et al (2019) Exploring the GDB-13 chemical space using deep generative models. J Cheminform 11:20. https://doi.org/10.1186/s13321-019-0341-z

7. Awale M, Reymond JL (2015) Similarity Mapplet: Interactive Visualization of the Directory of Useful Decoys and ChEMBL in High Dimensional Chemical Spaces. J Chem Inf Model 55:1509–1516. https://doi.org/10.1021/acs.jcim.5b00182

8. Nguyen KT, Blum LC, Van Deursen R, Reymond JL (2009) Classification of organic molecules by molecular quantum numbers. ChemMedChem 4:1803–1805. https://doi.org/10.1002/cmdc.200900317

9. Blum LC, Van Deursen R, Reymond JL (2011) Visualisation and subsets of the chemical universe database GDB-13 for virtual screening. J Comput Aided Mol Des 25:637–647.

https://doi.org/10.1007/s10822-011-9436-y
