# Supplementary material for: Randomized SMILES strings improve the quality of molecular generative models
Source: J Cheminform. 2019 Nov 21;11:71. doi: 10.1186/s13321-019-0393-0 (PMC6873550; doi:10.1186/s13321-019-0393-0)
Supplement: Supplementary file 2 — Additional file 2. Supplementary figures. [file 13321_2019_393_MOESM2_ESM.pdf]

Additional figures

Randomized SMILES strings improve the quality of molecular generative models

Josep Arús-Pous[§⊥*], Simon Johansson[§], Oleksii Prykhodko[§], Esben Jannik Bjerrum[§], Christian Tyrchan[‖], Jean-Louis Reymond[⊥], Hongming Chen[§], Ola Engkvist[§]

§ Hit Discovery, Discovery Sciences, R&D, AstraZeneca Gothenburg, Sweden

‖ Medicinal Chemistry, BioPharmaceuticals Early RIA, R&D, AstraZeneca, Gothenburg, Sweden

⊥ Department of Chemistry and Biochemistry, University of Bern, Freiestrasse 3, 3012 Bern, Switzerland.

* Corresponding author: josep.arus@dcb.unibe.ch

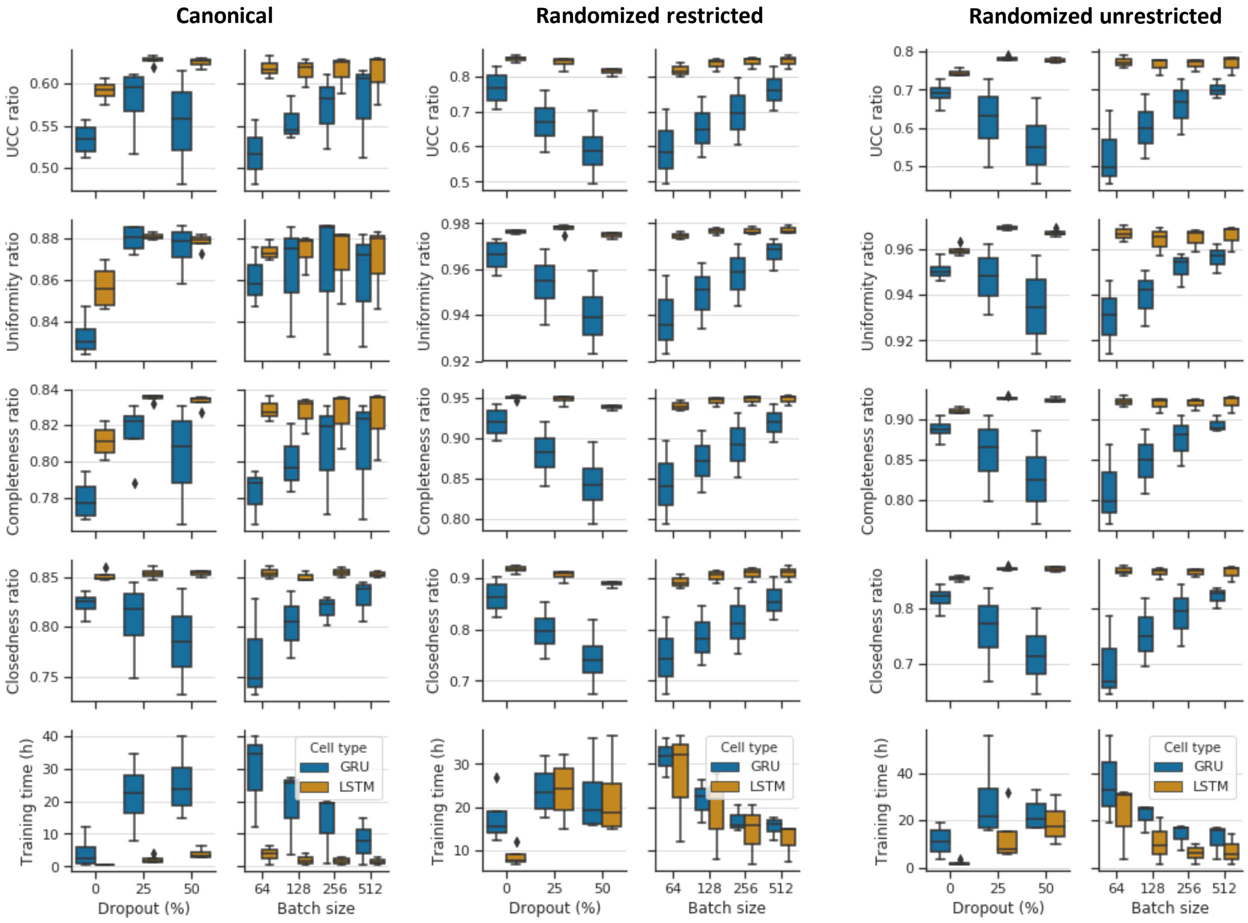

Figure S1: Boxplots of the four ratios (see methods) and the training time with all samples binned by dropout (first) and batch size (second) of the canonical (left), the randomized restricted (middle) and randomized unrestricted (right) SMILES variants. To account for the difference between cell types, for each bin the values are further separated between models using GRU and LSTM.

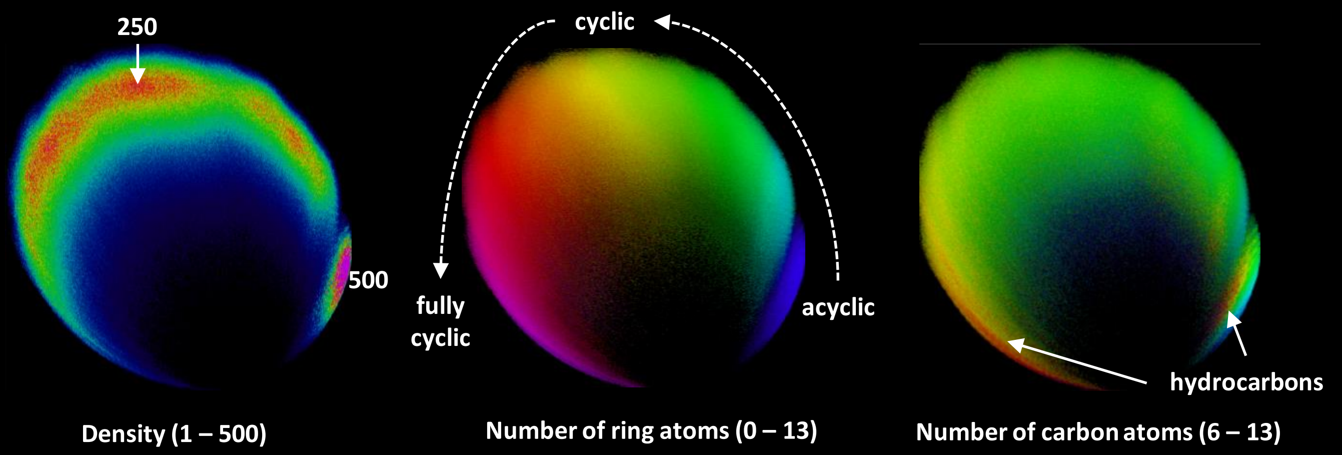

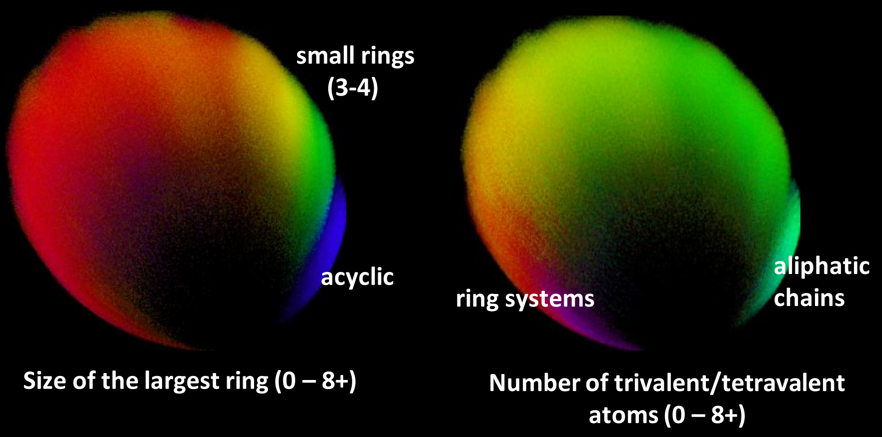

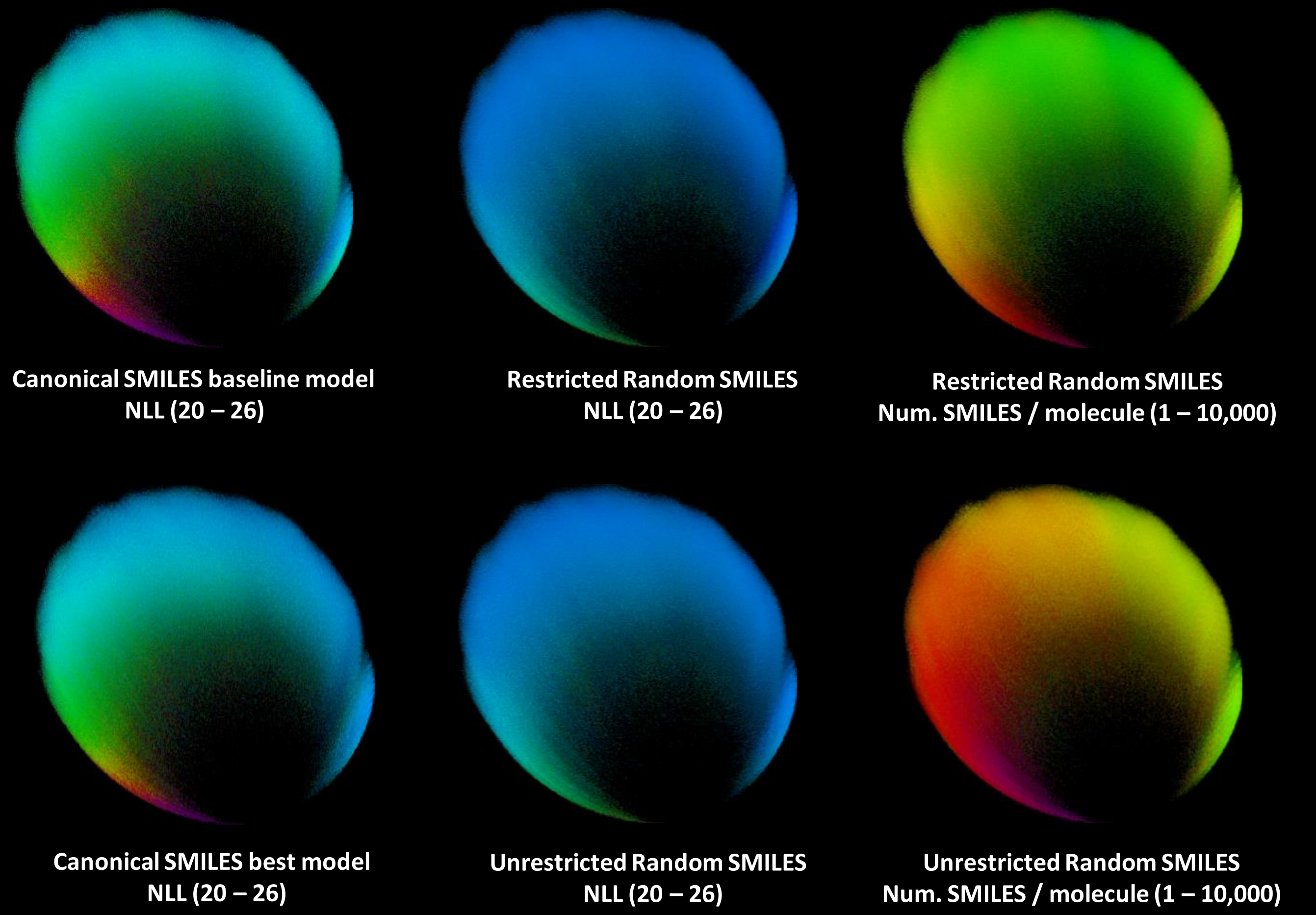

Figure S2: Similarity maps created with the MQN fingerprint (see methods) coloured by different descriptors. Each pixel is a group of molecules similar to each other and its colour depicts the average value for each pixel in the following order: blue, cyan, green, yellow, orange, red and magenta. The first map shows the density (how many molecules/pixel). The rest in the first two rows are coloured with physicochemical descriptors. The last two rows are coloured by the Negative Log-Likelihood (NLL) of the best models trained with a 1 million molecule set from GDB-13 with different SMILES variants. The last two on the right show the distribution of random SMILES / molecule in logarithmic scale for each of the variants.

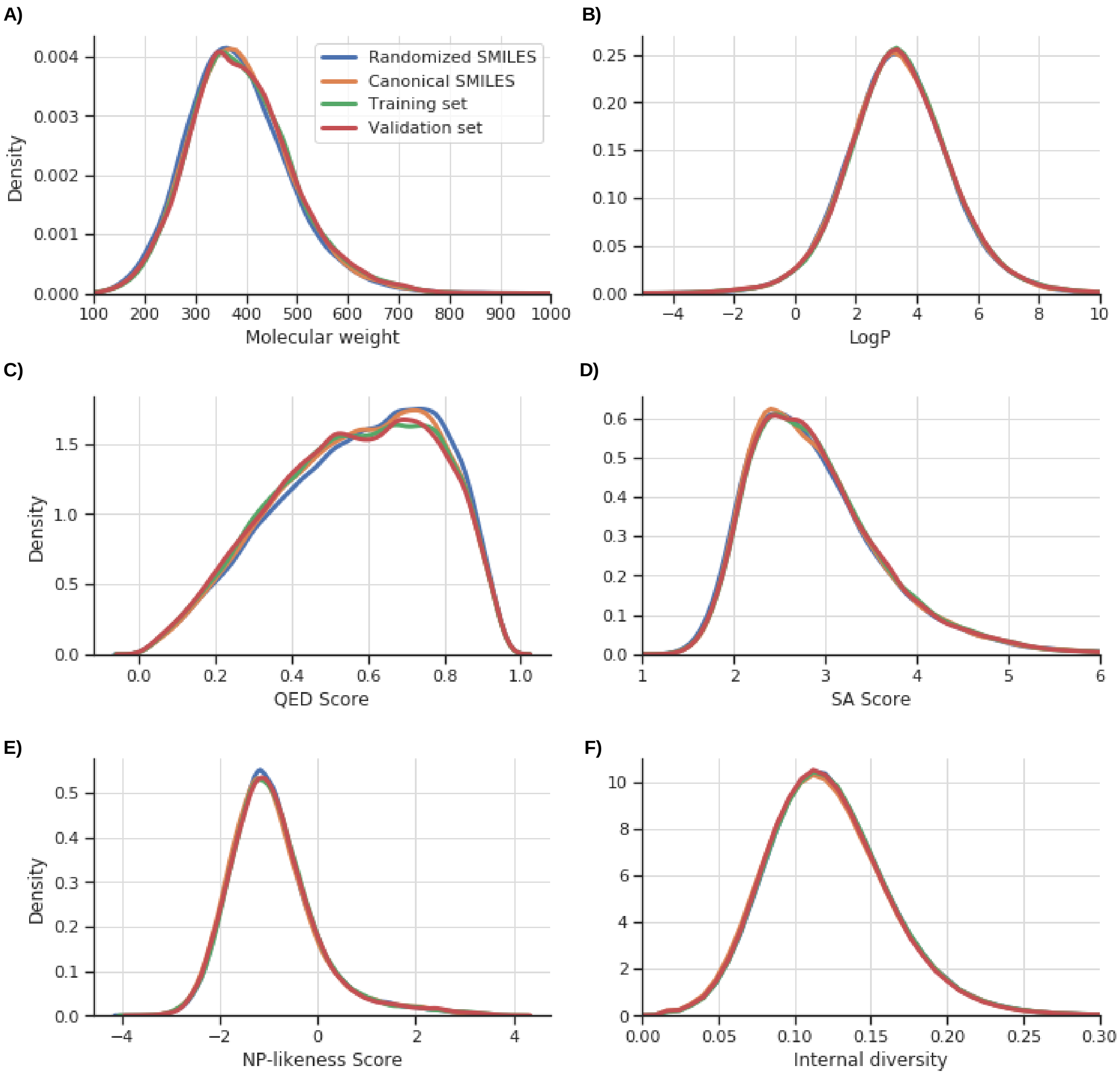

Figure S3: Kernel Density Estimates (KDEs) of various metrics and physicochemical descriptors for four samples of 50,000 molecules from the training set, validation set, best ChEMBL randomized and canonical SMILES models. **A)** Molecular weight **B)** Octanol-Water partition coefficient (LogP) **C)** Quantitative Estimate of Drug-likeness (QED) score. Measures how drug-like are the molecules (higher is better) **D)** Synthetic Accessibility (SA) Score. Assesses how synthesizable are the molecules (lower is better). **E)** Natural Product-likeness (NP) Score. Categorizes the drugs between synthetic-like (-4, 0), drug-like (-3, 2) and natural product-like (0, 4). **F)** Internal diversity. ECFP4 (hashed at 1024 bits) was obtained for a sample of 10,000 molecules of each set and pairwise Tanimoto similarity was performed and plotted. This metric shows how different are the molecules between each other. Notice that the x axis is cropped to (0, 0.3) from the full (0, 1) range.

DB	*Set*	*SMILES*	*Layers*	*Dimensions*	*Dropout (%)*	*Batch size*
GDB-13	1M	Canonical	3	512	25	64
		Rand. unr.	3	512	25	64
		Rand. unr. w.o.	3	512	25	128
		Rand. rest.	3	512	0	512
		Rand. rest. w.o.	3	512	25	256
		DS branch	3	512	25	128
		DS rings	3	512	25	128
		DS both	3	512	25	256
	10K	Canonical	3	384	50	8
		Rand. Rest.	3	384	50	32
	1K	Canonical	3	192	50	4
		Rand. Rest.	3	256	50	16
ChEMBL	1.5M	Canonical	3	512	25	128
		Rand. Rest.	3	512	0	128

Figure S4: A table with the hyperparameters of the best model for each dataset and SMILES type. All models were trained with LSTM cells.
